# Life-Threatening Hypercalcemia due to Graves' Disease and Concomitant Adrenal Failure: A Case Report and Review of the Literature

**DOI:** 10.1155/2015/684648

**Published:** 2015-03-24

**Authors:** Hande Mefkure Ozkaya, Fatma Ela Keskin, Ozlem Asmaz Haliloglu, Tugba Elif Senel, Pinar Kadioglu

**Affiliations:** Endocrinology and Metabolism, Cerrahpasa Medical School, University of Istanbul, 34098 Istanbul, Turkey

## Abstract

A 47-year-old woman presented with the complaints of nausea, vomiting, and weight loss. She had a history of bilateral surrenalectomy due to Cushing's syndrome. On examination she had tachycardia and orthostatic hypotension. Laboratory examinations revealed hypercalcemia and suppressed parathyroid hormone levels. She also had thyrotoxicosis due to Graves' disease. The investigations to rule out a malignancy were negative. With steroid, zoledronic acid, and antithyroid drug treatment her symptoms were resolved and calcium level was normalized. This case highlights the importance of recognizing thyrotoxicosis and concomitant adrenal failure as a possible cause of severe hypercalcemia.

## 1. Introduction

Severe or life-threatening hypercalcemia is, in most of the cases, either caused by neoplastic disease or primary hyperparathyroidism [1]. However there are some other rare forms of hypercalcemia that can represent a diagnostic challenge to the physicians. Hyperthyroidism is known to cause a relatively mild hypercalcemia and elevated serum calcium levels are seen in approximately one-fifth of the cases [2]. An increase in bone turnover and mobilization of calcium from bone into the circulation are the main mechanisms responsible for the hypercalcemia associated with hyperthyroidism [3]. Concomitant conditions like hypocortisolism may aggravate hypercalcemia seen during hyperthyroidism and thyroid hyperfunction per se may induce a relative hypocortisolemic state, thus exacerbating further the degree of calcium elevation [4]. We herein report a case of 47-year-old woman presenting with life-threatening hypercalcemia due to Graves' disease accompanied by acute adrenal failure.

## 2. Case Presentation

A 47-year-old woman was admitted to our clinic with the complaints of nausea, intermittent vomiting, constipation, fatigue, and palpitation. She also had progressive weight loss of 10 kg since last 5 months. She had a history of bilateral surrenalectomy 3 years ago due to Cushing's syndrome associated with adrenocorticotropic hormone (ACTH) independent macronodular adrenal hyperplasia. The patient was taking prednisolone 7.5 mg and fludrocortisone 0.1 mg orally once a day. Her family history was significant for breast cancer in her mother, gastric cancer in her aunt, and coronary artery disease in her uncle. Physical examination revealed tachycardia, presence of lid lag, warm skin, and orthostatic hypotension. The thyroid gland was enlarged and had a bruit. Pertinent laboratory values included a serum thyroid stimulating hormone (TSH) of 0.005 mIU/L (normal, 0.4 to 4.2) with a free T4 value of 7.7 ng/dL (normal, 0.93 to 1.7) and free T3 value of 16 pg/mL (normal, 2 to 4.4). Thyroglobulin antibody (Anti TG) and thyroid receptor antibody (TRAB) were elevated (anti-TG 718 IU/mL (normal, 0 to 115) and TRAB 14.91 U/mL (normal, 0 to 1.1)). Thyroid scan showed bilateral mildly increased, diffuse, and homogenous uptake in the thyroid gland. Radioactive iodine uptake was calculated to be 19% at two hours and 39% at 24 hours. Thyroid ultrasonography revealed an enlarged and heterogeneous thyroid gland. The patient's serum calcium level was 16 mg/dL (normal, 8.4 to 10.2), serum phosphorus was 3.9 mg/dL (normal, 2.5 to 4.5), albumin level was in the normal range, and intact parathyroid hormone (iPTH) was found to be suppressed to the level of 7.99 pg/mL (normal, 15 to 65). The 25-OH vitamin D was slightly decreased (21 ng/dL (normal, 30 to 50)) and serum alkaline phosphatase was normal. Serum creatinine was in the normal range, serum sodium was 136 mmol/L (normal, 136 to 145), and potassium was 5.1 mmol/L (normal, 3.5 to 5.1). The electrocardiogram showed sinus tachycardia at a rate of 120 beat per minute (bpm) and shortened QT interval. In the lights of these initial physical and laboratory investigations, the diagnosis of thyrotoxicosis complicated by hypercalcemia and acute adrenal failure was confirmed. Emergent treatment with intravenous saline, methylprednisolone 20 mg intravenously 3 times a day, methimazole 10 mg orally 3 times a day, and propranolol 20 mg orally 2 times a day was started. Fludrocortisone at a dose of 0.1 mg orally was continued. After infusion of 2 liters of isotonic saline, furosemide was added to the therapy and saline infusion was continued at rate of 250 mL/hour. Zoledronic acid at a dose of 4 mg intravenously was also given to the patient. On the third day of the admission, the patient's calcium level was 9.5 mg/dL, and saline and furosemide infusion were stopped. The free T4 level was 4.67 ng/dL and free T3 level was 5.98 pg/mL. On the fourth day of admission, the patient complained of acute onset right upper quadrant pain. The laboratory examinations revealed that transaminase levels were elevated to approximately three times of the upper limit of normal. As her initial transaminase values were within the normal range, hepatotoxicity due to the methimazole administration was suspected and the drug was stopped. Hepatobiliary ultrasonography was consistent with acute hepatitis and viral serologic examinations were negative. After three days while off methimazole serum transaminase values were normalized and the patient had no more pain in the right upper quadrant, we started the patient on cholestyramine 8 g orally 3 times a day, lithium 600 mg orally 2 times a day, increased the propranolol dose to 40 mg 3 times a day, and continued methylprednisolone at the same dose. The investigations to rule out a possible malignancy including thorax and abdominal computed tomography (CT), bone marrow examination, gastroscopy, bone scan, gynecologic and mammographic examinations, and PET-CT were all negative. After being euthyroid the patient received 10 millicuries (mCi) radioactive iodine (RAI). The patient was discharged from the hospital with a steroid taper schedule aiming at 8 mg dose decrease every 5 days until the final dose of 7.5 mg per day of prednisolone was reached. Three months later, the patient was readmitted to the hospital with similar complaints and laboratory examinations revealed a mild thyrotoxicosis, mild hypercalcemia, hyponatremia, and hyperpotassemia. The clinical picture and laboratory findings of the patient were less remarkable at that time as she had increased the steroid dose one week before her admission. She was using prednisolone 5 mg orally three times a day and these supraphysiological doses of steroid may have potentially prevented the development of more severe adrenal failure and thus hypercalcemia. An additional dose of 15 mCi RAI was administered to the patient. She became hypothyroid and was started on levothyroxine replacement therapy 3 months later. At her last follow-up visit, she was euthyroid and normocalcemic and her iPTH was 41 pg/mL.

## 3. Discussion

Hypercalcemia, defined as serum calcium levels above 10.5 mg/dL, is a usually mild and well-tolerated clinicopathologic entity. Primary hyperparathyroidism is the most common cause of hypercalcemia seen in ambulatory patients and is usually asymptomatic. Hypercalcemia associated with malignancy tends to be more frequently symptomatic and is the most common cause of hypercalcemia in hospitalized patients. Together, these two entities constitute 90% of the cases of hypercalcemia. On the other hand, more severe calcium elevations, either de novo or as an exacerbation of preexisting mild hypercalcemia, can lead to life-threatening hypercalcemic crisis which require urgent medical treatment to prevent vital organ dysfunction [5].

We presented here a patient with life-threatening and symptomatic hypercalcemia which was attributed to the combined effect of hyperthyroidism and hypocortisolism after extensive research to rule out other possible causes. However these conditions are rarely reported to be associated with markedly elevated serum calcium levels and in general they present with mild hypercalcemia unless there are some other aggravating factors as being presented in our case [6, 7].

Thyroid hormones are known to increase bone resorption and formation with predominance of the bone resorption. That is also the main mechanism leading to elevated serum calcium levels seen in hyperthyroid patients [8]. Increased interleukin-6 levels and hyperadrenergic state induced by thyrotoxicosis are also implicated in hypercalcemia [9]. Indeed thyroid hyperfunction is usually associated with mild hypercalcemia. In a large series of hyperthyroid patients when corrected for the hyperthyroid state, serum calcium level greater than 10.6 mg/dL was found in only 5.7% of the study population. There was no case of symptomatic hypercalcemia with only one case with coincident primary hyperparathyroidism having serum calcium level greater than 11.2 mg/dL [6]. As hyperthyroidism is associated with increased bone turnover, high levels of serum alkaline phosphatase mostly of bone origin are usually detected in thyrotoxic patients. Interestingly, they tend to increase further with therapy which is suggestive of decreased bone resorption and increased bone formation with deposition of bone mineral after antithyroid treatment [8]. In our case, although we did not measure the bone isoenzyme which was more likely to be elevated, total alkaline phosphatase level was initially normal but tended to rise following normalization of thyroid hormones with therapy. It can be postulated that calcium stores were replenished after therapy and that phenomenon led to increased bone formation. Also previous administration of a bisphosphonate might have been a possible confounding factor preventing further elevations of alkaline phosphatase [10].

As with thyrotoxicosis, both primary and secondary adrenal failure have been associated with hypercalcemia although the clinical picture and degree of hypercalcemia are usually more severe in the former [7]. Increased renal calcium absorption, hypovolemia, and calcium release from bone into the circulation are the main mechanisms responsible for the hypercalcemia seen in cortisol deficiency [7, 11]. Cortisol exerts antagonistic effects to thyroid hormones on bone, increases renal calcium excretion, and decreases gastrointestinal calcium absorption. On the other hand, thyroid hormones appear necessary for the development of hypercalcemia encountered in the setting of adrenal failure as it was shown that, in adrenalectomized dogs, severe hypercalcemia which developed following adrenalectomy was prevented by simultaneous thyroidectomy [7, 11]. Also initiation of levothyroxine therapy in a case with Addison's disease was associated with severe hypercalcemia [11]. As thyroid hormones are known to increase cortisol clearance [7], we can postulate that in our case sudden onset hyperthyroidism induced a relative hypocortisolemic state leading to the further aggravation of the hypercalcemia. In other words, combined effects of acute adrenal failure which developed following thyrotoxicosis and hyperthyroidism per se may act in synergistic way and worsen the hypercalcemic state, as being presented in our case.

In the English literature, there have been thirteen case reports of thyrotoxicosis complicated with hypercalcemia and adrenal failure [7, 12–21]. The general characteristics, biochemical findings, and clinical course of patients are summarized in Tables 1 and 2. Nine out 13 patients were female and 4 were male. In these cases, adrenal failure was primarily due to secondary hypopituitarism, lymphocytic hypophysitis being the most common underlying etiology. There were also one case of idiopathic Addison's disease, one case with unilateral adrenalectomy who developed adrenal insufficiency while tapering the steroid dose, one case with panhypopituitarism secondary to ruptured Rathke's cleft cyst, one case with a hypothalamic tumor, another case with macroprolactinoma, and one case with isolated ACTH deficiency. The thyrotoxicosis was most commonly associated with silent or postpartum thyroiditis. There were also 3 cases with Graves' disease and one case with iodine induced thyrotoxicosis. Serum calcium levels were ranging from 12 to 16.3 mg/dL. In the majority of the cases, hypercalcemia and debilitating clinical state of the patients were improved within a few days of steroid and intravenous fluid administration. Six patients were treated with antithyroid drugs; one of them received also intravenous bisphosphonate infusion as in our case. Our patient presented with a very high calcium level and needed combined treatment with steroids, intravenous fluids, and zoledronic acid in addition to propranolol and antithyroid drugs which were stopped later due to the hepatotoxicity. Although the patient's serum calcium level normalized rapidly, she needed high dose of steroid, propranolol, lithium, and cholestyramine to control the thyrotoxicosis and she was still hyperthyroid despite the first course of radioactive iodine treatment. However her calcium level was just slightly elevated as she was given supraphysiological doses of steroid pointing out to the role of steroids in the control of hypercalcemia encountered in the setting of thyrotoxicosis and adrenal failure.

As a conclusion, by presenting this case and previous reports we discussed the occurrence of hypercalcemia which can be, not infrequently, life-threatening in the setting of concomitant thyrotoxicosis and adrenal failure. It seems that thyroid hormones and glucocorticoids play important regulatory role in the calcium homeostasis by mechanisms still not fully understood but independent of parathyroid hormone. In the clinical setting, this case illustrates the importance of considering the possibility of coexisting adrenal failure as an aggravating factor in a patient with thyrotoxicosis who presents with severe hypercalcemia.

## Figures and Tables

**Table 1:** Clinical and biochemical findings of the reported cases.

Case	A/S	TE	AFE	Ca-P-PTH NR	(f)T3-(f)T4-TSH NR	Cortisol-ACTH NR	R
1	31/F	ST	LH	16.3 mg/dL-5.4 mg/dL-ND	ND/12 *µ*g/dL/ND	ND-ND	[12]
				(8.5–9.7)-ND-ND	ND/(4–11)/ND	ND-ND	

2	28/F	ST	LH	12.1 mg/dL-6.6 mg/dL-26 pg/mL	ND/20.2 *µ*g/dL/ND	2 *µ*g/dL-15 pg/mL	[13]
				(8.4–10.2)-(2.5–4.5)-(20–70)	ND/(5.0–11.5)/ND	ND-(0–23)	

3	32/F	ST	LH	14.8 mg/dL-ND-ND	ND-14 *µ*g/dL-<0.1	<2 *µ*g/dL-24 pg/mL	[14]
				ND-ND-ND	ND-(5–12.5)-(0.5–6.0)	ND-(<120)	

4	43/F	GD	AD	14.2 mg/dL-ND-ND	4.5 ng/mL-17.2 *µ*g/dL-ND	<5 *µ*g/dL-ND	[15]
				ND-ND-ND	(0.84–2.1)-ND-ND	ND-ND	

5	29/F	ST	LH	3.12 mmol/L-0.73 mmol/L (0.75–1.35)-<1.5 mol/L	ND-30 pmol/L-0.2 mU/L	35 nmol/L-7 ng/L	[16]
				(2–2.5)-(0.75–1.35)-(1.6–6.9)	ND-(10–20)-ND	ND-(10–60)	

6	41/F	ST	LH	12 mg/dL-ND-<1.5 pmol/L	1.95 pg/mL-1.76 ng/dL-<0.02 mU/L	1.5 *µ*g/dL-ND	[17]
				ND-ND-(2–5)	(2.0–5.6)-(0.85–2.3)-(0.3–4.8)	ND-ND	

7	54/M	GD	SHP^*^	3.4 mmol/L-ND-ND	431^**^/9.8^†^/ND	ND-ND	[17]
				ND-ND-ND	(59–127)-(0.9–2.3)-ND	ND-ND	

8	24/M	GD	SHP^‡^	13.2 mg/dL-5 mg/dL-<5 mg/dL	8.7 pg/mL-3.6 ng/dL-<0.1 mU/L	1.9 *µ*g/dL-25.9 pg/mL	[4]
				(8.5–10.5)-ND-(10–65)	(2.2–4.5)-(0.7–1.8)-(0.35–4.0)	ND-ND	

9	45/M	ST	LH	Ca: 3.56 mmol/L-1.51 mmol/L-<8 ng/L	7 nmol/L-75 ng/dL-<0.05 mU/L	50 nmol/L-10 ng/L	[18]
				ND-(0.75–1.35)-(10–65)	(1.0–2.6)-(8–27)-(0.15–3.5)	ND-ND	

10	36/F	ST	LH	3.35 mmol/L-1.3 mmol/L-<0.5 *µ*g/L	30 pmol/L-82 pmol/L-<0.03 mU/L	<3 nmol/L-1 pmol/L	[19]
				(2.2–2.56)-ND-ND	(3.8–6.6)-(12–25)-ND	ND-ND	

11	64/F	IHH	IAD	3.29 mmol/L-1.78 mmol/L-0.9 pmol/L	6.2 pmol/L-20.1 pmol/L-0.01 mU/L	ND-ND	[7]
				(2.1–2.6)-(0.8–1.5)-(1.1–7.7)	(1–5.2)-(9–19)-ND	ND-ND	

12	53/F	ST	UA	12.4 mg/dL-4.7 mg/dL-10 pg/mL	10.4 pg/mL-3.99 ng/dL-0.015 mU/L	8.8 *µ*g/dL-23.3 pg/mL	[20]
				ND-ND-ND	(2.4–4.0)-(0.94–1.6)-(0.38–4.3)	ND-ND	

13	32/M	ST	SHP^§^	12 mg/dL-5.5 mg/dL-2 pg/mL	4.48 ng/mL-5.47 ng/dL-<0.01 mU/L	5.1 *µ*g/dL-24.6 pg/mL	[21]
				(8.4–10.2)-(2.5–5.5)-(5–99)	(0.6–1.6)-(0.8–1.54)-(0.35–5.5)	ND-ND	

PC	47/F	GD	BA	16 mg/dL-3.9 mg/dL-7.99 pg/mL	ND/7.7 ng/dL/0.005 mIU/L	0.533 *µ*g/dL-ND
				(8.4–10.2)-(2.5–4.5)-(15–65)	ND/(0.93–1.7)/(0.4–4.2)	(5–23)-ND

A, age; S, sex; TE, thyrotoxicosis etiology; AFE, adrenal failure etiology; Ca, calcium; P, phosphorus; PTH, parathyroid hormone; NR, normal range; (f)T3, free or total triiodothyronine; (f)T4, free or total thyroxin; TSH, thyroid stimulating hormone; ACTH, adrenocorticotropic hormone; F, female; M, male; ST: silent thyroiditis; LH: lymphocytic hypophysitis; ND, not determined; GD, Grave's disease; AD, Addison's disease; SHP, secondary hypopituitarism; IHH, iodine induced hyperthyroidism; IAD, isolated ACTH deficiency; UA, unilateral adrenalectomy; PC, present case; BA, bilateral adrenalectomy.

^*^Due to macroprolactinoma resection; ^**^free triiodothyronine index, ^†^free thyroxin index; ^‡^due to hypothalamic tumor; ^§^due to ruptured Rathke's cleft cyst.

**Table 2:** Signs, symptoms, and treatment regimens of the reported cases.

Case	Signs/symptoms	Treatment	R
1	Anorexia, weight loss, fasting hypoglycemia, and mental aberrations	IVH, Di	[12]
2	Weakness, anorexia, orthostatic dizziness, failure to lactate, and weight loss	BB, C	[13]
3	Myalgia, anorexia, nausea, vomiting, weight loss, and failure to lactate	IVH, C	[14]
4	Lethargy, anorexia, vomiting, and weight loss	IVH, C, AT, RAI	[15]
5	Fatigue, weakness, nausea, vomiting, myalgia, arthralgia, and weight loss	C	[16]
6	Lethargy, myalgia, arthralgia, myopathy, nausea, vomiting, anorexia, weight loss, and failure to lactate	C, AT	[17]
7	Confusion, tachycardia, and dehydration	C, RAI	[17]
8	Disturbed mental state, severe nausea, vomiting, increased thirst, polyuria, and weight loss	AT, C, DDAVP	[4]
9	Anorexia, diarrhea, night sweats, myalgia, and weight loss	IVH, BP, AT, C	[18]
10	Headache, anorexia, weakness, fever, tachycardia, mental changes, and failure to lactate	C, IVH, KI, AT, BB	[19]
11	Nausea, vomiting, fever, and lethargy	C	[7]
12	Anorexia, nausea, vomiting, and severe dehydration	C, IVH	[20]
13	Vomiting and diarrhea	IVH, BB, C	[21]
PC	Vomiting, nausea, constipation, and weight loss	IVH, C, Di, BP, AT, BB	

R, reference; IVH, intravenous hydration; Di, diuretic; BB; beta blocker; C, corticosteroid; AT, antithyroid drugs; RAI, radioactive iodine treatment; DDAVP, 1-deamino-8-D-arginine vasopressin; BP, bisphosphonate; KI, potassium iodide; PC, present case.
